# Graphitic Carbon Quantum Dots Modified Nickel Cobalt Sulfide as Cathode Materials for Alkaline Aqueous Batteries

**DOI:** 10.1007/s40820-019-0355-0

**Published:** 2020-01-04

**Authors:** Yirong Zhu, Jingying Li, Xiaoru Yun, Ganggang Zhao, Peng Ge, Guoqiang Zou, Yong Liu, Hongshuai Hou, Xiaobo Ji

**Affiliations:** 1 College of Metallurgy and Material Engineering, Hunan University of Technology, Zhuzhou, 412007 People’s Republic of China; 2 College of Chemistry and Chemical Engineering, Central South University, Changsha, 410083 People’s Republic of China; 3 State Key Lab of Powder Metallurgy, Central South University, Changsha, 410083 People’s Republic of China

**Keywords:** Energy storage, Alkaline aqueous batteries, Carbon quantum dot, Nickel cobalt sulfide

## Abstract

Carbon quantum dots (CQDs) as a new class of emerging materials have gradually drawn researchers’ concern in recent years. In this work, the graphitic CQDs are prepared through a scalable approach, achieving a high yield with more than 50%. The obtained CQDs are further used as structure-directing and conductive agents to synthesize novel N,S-CQDs/NiCo_2_S_4_ composite cathode materials, manifesting the enhanced electrochemical properties resulted from the synergistic effect of highly conductive N,S-codoped CQDs offering fast electronic transport and unique micro-/nanostructured NiCo_2_S_4_ microspheres with Faradaic redox characteristic contributing large capacity. Moreover, the nitrogen-doped reduced graphene oxide (N-rGO)/Fe_2_O_3_ composite anode materials exhibit ultrahigh specific capacity as well as significantly improved rate property and cycle performance originating from the high-capacity prism-like Fe_2_O_3_ hexahedrons tightly wrapped by highly conductive N-rGO. A novel alkaline aqueous battery assembled by these materials displays a specific energy (50.2 Wh kg^−1^), ultrahigh specific power (9.7 kW kg^−1^) and excellent cycling performance with 91.5% of capacity retention at 3 A g^−1^ for 5000 cycles. The present research offers a valuable guidance for the exploitation of advanced energy storage devices by the rational design and selection of battery/capacitive composite materials.

**Electronic supplementary material:**

The online version of this article (10.1007/s40820-019-0355-0) contains supplementary material, which is available to authorized users.

## Highlights

- The scalable graphitic carbon quantum dots (CQDs) are obtained with a high yield of more than 50%.
- The CQDs are utilized to synthesize novel NS-CQDs/NiCo_2_S_4_ composite which shows enhanced electrochemical properties.
- The assembled novel alkaline aqueous battery delivers superior electrochemical performances.

**Electronic supplementary material:**

The online version of this article (10.1007/s40820-019-0355-0) contains supplementary material, which is available to authorized users.

## Graphical abstract

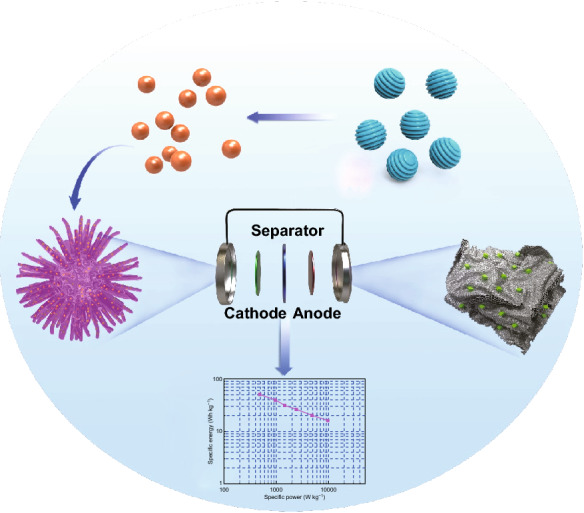

## Introduction

Carbon quantum dots (CQDs), as a novel type of zero-dimensional (0D) carbon materials with ultrasmall sizes less than 10 nm, have been extensively applied in fluorescence, bioimaging, biosensing, photocatalysis and photovoltaics owing to their unique advantages such as quantum confinement and edge effects [1–4]. In particular, the application of CQDs in energy storage has gradually attracted researchers’ attention in recent years [5–7]. In the early research, the reduced carbon quantum dots (rCQDs)/RuO_2_ composites have been obtained as ultrafast supercapacitor electrode materials for the first time, where rCQDs are utilized as conductive agents to boost the electrical conductivity of electrode materials, enhancing the supercapacitive properties, especially rate capability [8]. Since this reported pioneering work, more researches about such CQDs-based energy storage materials as supercapacitors, lithium/sodium ion batteries and fuel cell have been explored [9–14], which indicates that these upcoming CQDs-based electrode materials present a great prospect as superior energy storage materials. Nevertheless, the utilization of CQDs as electrode materials is much less reported compared to the conventional carbon materials; hence, it shows a great potential to further explore them.

Currently, the methods for the synthesis of CQDs are usually classified into “top-down” and “bottom-up” types. The former is usually breaking down large-size carbon materials into CQDs, including arc discharge, electrochemical exfoliation, hydrothermal technique and chemical oxidation synthesis [15–18], whereas the latter is usually preparing CQDs from molecular precursors, such as combustion/thermal decomposition method, supported synthesis and microwave pyrolysis synthetic route [19–21]. However, these approaches for the synthesis of CQDs still face major challenges, such as a low yield (usually lower than 10%), complicated processes, poor reproducibility and electronic conductivity [1]. In our previous study, graphitic CQDs were obtained via chemical oxidation synthesis route utilizing nano-graphite powders (~30 nm) as precursors, and a relatively low yield of less than 20% was acquired [8, 10]. Hou et al. obtained non-graphitic CQDs through a facile and scalable solution growth method by mixing NaOH and acetone together, and an ultrahigh yield with gram level was achieved. However, the resulted CQDs presented low electronic conductivity, leading to the poor electrochemical properties [12]. From the perspective of practical application for energy storage electrode materials, it is crucially important to develop a scalable approach for synthesizing CQDs with a high yield, low cost and high electrical conductivity.

Besides, another major challenge for CQDs is that they are apt to aggregate and stack together, which results in a rapid decrease in their specific surface areas and kinetic behaviors, thus worsening the electrochemical properties of CQDs [22]. These obstacles above prevent their utilization as excellent energy storage electrode materials. The most effective method to solve the aggregation is to employ them as composite materials. Recently, ternary metal oxides/sulfides have attracted extensive interests because of their higher electrical conductivity and richer electrochemical activity than those of the corresponding binary metal oxides/sulfides, which have been explored as potential cathode materials for alkaline aqueous batteries [23–28]. Among them, NiCo_2_S_4_ is seen as the most prospective candidate for future utilization in such energy storage as supercapacitors and lithium ion batteries. To further enhance its comprehensive electrochemical performances, an effective way is to design various unique micro-/nanostructured NiCo_2_S_4_, and the enhanced electrochemical properties have been reported by researchers in the literature [29–33]. Another good way is to introduce carbon materials to prepare the composite electrode materials, leading to the enhanced electrochemical properties to some extent. Currently, the most reported carbon materials are graphene and carbon nanotubes, demonstrating decent electrochemical properties, but their high cost seriously prevents the practical application [34, 35]. Therefore, it is very emergent to exploit novel and cheap carbon materials for the fabrication of superior composite electrode materials.

As discussed above, the scalable graphitic CQDs were first synthesized with a high yield of more than 50% utilizing highly conductive Super P powders as precursors. Then, the CQDs were employed as both structure-directing and conductive agents to prepare new N,S-codoped CQDs-decorated urchin-like NiCo_2_S_4_ microspheres for the first time. In comparison with the pure NiCo_2_S_4_ electrode, the N,S-CQDs/NiCo_2_S_4_ composite electrode, when for the first time used as cathode for alkaline aqueous batteries, exhibits enhanced electrochemical properties with a high specific capacity of 124.4 mAh g^−1^ at 2 A g^−1^, exceptional capacity retention of 77.3% at 50 A g^−1^ in comparison with 2 A g^−1^ and excellent cycling property with 97.9% of initial capacity retention at 3 A g^−1^ for 5000 cycles. Meanwhile, the N-rGO-wrapped prism-like Fe_2_O_3_ hexahedrons composite anode materials are obtained, which manifest substantially enhanced electrochemical performances with an ultrahigh specific capacity (249.6 mAh g^−1^ at 1 A g^−1^) as well as greatly enhanced rate property and cycling life compared with those of the pristine Fe_2_O_3_ electrode. A novel N,S-CQDs/NiCo_2_S_4_//N-rGO/Fe_2_O_3_ alkaline aqueous battery fabricated by these materials achieves a specific energy (50.2 Wh kg^−1^) and ultrahigh specific power (9,700 W kg^−1^) with excellent cycle property (91.5% of capacity retention for 5000 cycles). The encouraging results display the enormous potential of the new alkaline aqueous battery as highly promising energy storage system for potentially practical applications.

## Experimental Section

### Synthesis of CQDs

CQDs were obtained by a scalable chemical oxidation approach. Super P powders (1 g, purchased from Sigma-Aldrich) were first added into concentrated H_2_SO_4_/HNO_3_ mixed solution (80 mL, v/v, 3:1) and then sonicated for 1 h. The resulting mixture was refluxed for 24 h. Subsequently, the mixture was further neutralized with Na_2_CO_3_·10H_2_O, and the pH was controlled at 7.0. After further dialysis and vacuum-drying, the final CQDs powders were obtained.

### Synthesis of N,S-CQDs/NiCo_2_S_4_ Composite Materials

The N,S-CQDs/NiCo_2_S_4_ composite materials were synthesized by a one-step hydrothermal approach. Firstly, CQDs powders (22.3 mg) were dissolved in H_2_O/ethanol mixed solution (60 mL, v/v, 1:1) and agitated for 10 min. Subsequently, NiCl_2_·6H_2_O (0.06952 g) and CoCl_2_·6H_2_O (0.13919 g) were added into the obtained mixture and stirred for 20 min. Then, thioacetamide (0.13185 g) was further introduced into the above mixture. After continual agitating for another 20 min, the as-resulted mixed solution was transferred into a stainless steel autoclave lined with PTFE (100 mL) and hydrothermally treated at 180 °C for 24 h. The products were obtained by alternate centrifuging with H_2_O and ethanol for three times and further vacuum-dried overnight at 70 °C. For comparison, the pure NiCo_2_S_4_ was synthesized by the similar method only without adding CQDs powders in this preparation process.

### Synthesis of N-rGO/Fe_2_O_3_ Composite Materials

The N-rGO/Fe_2_O_3_ composite materials were prepared by a facile hydrothermal approach. Firstly, graphite oxide (GO, 40 mg, purchased from XFNANO) was dissolved in H2O solution (30 mL) and sonicated for 1 h. Then, FeCl3·6H2O (0.5406 g) was dissolved in H2O solution (15 mL) and stirred for 10 min. The as-resulted FeCl_3_ aqueous solution was added into the GO solution and sonicated for 30 min. Meanwhile, urea (0.6006 g) was dissolved in H2O solution (15 mL) and magnetically stirred for 10 min. Furthermore, the resulting urea solution was slowly introduced into the above mixture under strong agitation. After stirring for 30 min, the as-obtained mixture was transferred into a stainless steel autoclave lined with PTFE (100 mL) and subjected to hydrothermal reaction at 180 °C for 10 h. Lastly, the products were obtained by alternately centrifuging with H_2_O and ethanol for three times and further vacuum-dried overnight at 70 °C. For comparison, the pure Fe_2_O_3_ was synthesized by the similar method only in the absence of GO in this preparation process.

### Materials Characterization

X-ray diffraction (XRD) analysis was implemented on an X-ray diffractometer (Rigaku D/max 2550 VB^+^). Raman spectra were executed on a Raman spectrometer (Labram-010). X-ray photoelectron spectroscope (XPS) analysis was conducted on a photoelectron spectrometer (K-Alpha). FESEM images, EDS and elemental mappings were captured on a field emission scanning electron microscopy (FESEM, MIRA3). Transmission electron microscope (TEM) images, HRTEM images and SAED patterns were obtained on a transmission electron microscopy (JEM-2100F). TG curves were recorded from a thermal analysis instrument (NETZSCH STA449C). N_2_ adsorption/desorption isotherms were acquired on a BET specific surface area analyzer (BELSORP-miniII).

### Electrochemical Measurements

To fabricate the working electrodes for electrochemical tests, 70 wt% of electroactive material, 20 wt% of Super P and 10 wt% of PVDF with NMP as solvent were adequately mixed to obtain an uniform slurry. Afterward, the resulting slurry was dipped into Ni foam current collector, which was pretreated based on our previous work [10], and dried in a vacuum oven at 70 °C overnight. Finally, the as-fabricated electrodes were further pressed at 10 MPa. The electrochemical properties of the single electrode were conducted by employing a typical three-electrode cell with platinum foil, Hg/HgO and 2 M KOH solution as counter electrode, reference electrode and electrolyte, respectively. The alkaline aqueous coin battery (CR 2016) was further assembled by utilizing N,S-CQDs/NiCo_2_S_4_ as cathode, N-rGO/Fe_2_O_3_ as anode, glassy fibrous material as separator and 2 M KOH solution as electrolyte. The electrochemical tests such as CV, GCD and EIS for both single electrode and alkaline aqueous device were executed on an electrochemical workstation (CHI 760E).

## Results and Discussion

### Preparation Processes of the CQDs and the Composite Cathode and Anode

**Fig. 1:**
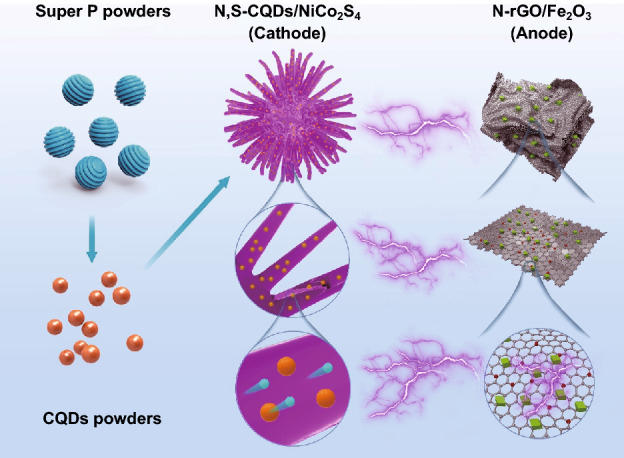
Schematic illustration of the synthetic processes of the CQDs, the N,S-CQDs/NiCo_2_S_4_ composite cathode and N-rGO/Fe_2_O_3_ composite anode

The synthetic processes of the graphitic CQDs, the N,S-CQDs/NiCo_2_S_4_ composite cathode and N-rGO/Fe_2_O_3_ composite anode are demonstrated in Fig. 1. First, the graphitic CQDs are prepared through a scalable mixed-acid chemical oxidation approach using the industrialized, cheap and highly conductive Super P powders as precursors. The obtained CQDs achieve a high yield of more than 50%, which is far higher than that prepared by most of previous work reported in the literature [1, 8, 10, 36, 37], demonstrating the great prospect for their potentially practical application. Afterward, the resulting CQDs are further employed as structure-directing and conductive agents to prepare novel N,S-codoped CQDs-decorated urchin-like NiCo_2_S_4_ microspheres composite cathode materials via a one-step hydrothermal approach, in which thioacetamide is used as precipitant to generate the NiCo_2_S_4_ and codoped agent to achieve nitrogen and sulfur codoping into the CQDs. In this process, the CQDs are not only utilized as structure-directing agents to induce the formation of nanorods-assembled urchin-like composite microsphere structure, but also served as conductive agents to improve the electrical conductivity of the composite cathode materials. Meanwhile, the N-doped rGO-wrapped prism-like Fe_2_O_3_ hexahedrons composite anode materials are prepared by a facile hydrothermal synthetic route, where urea serves as precipitant to generate the Fe_2_O_3_ and acts as doped agent to achieve nitrogen doping into the GO. The Fe_2_O_3_ hexahedrons are tightly wrapped by the N-rGO sheets, and the close contact between the Fe_2_O_3_ hexahedrons and the N-rGO sheets can improve the electrochemical kinetic behavior and structural stability of the composite anode materials.

### Physicochemical Characterization of CQDs

**Fig. 2:**
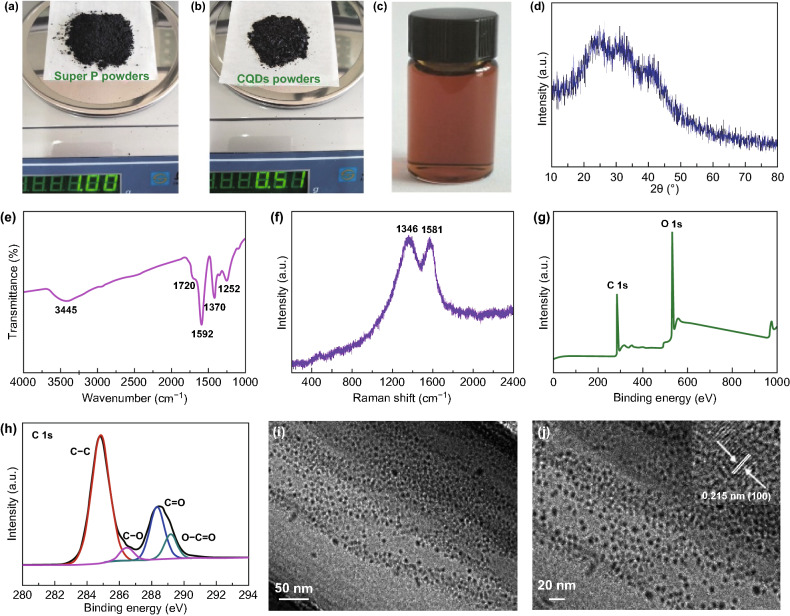
Digital photograph of **a** Super P powders, **b** CQDs powders, and **c** CQDs aqueous solution with a concentration of 1 mg mL^−1^. **d** XRD pattern, **e** FTIR spectroscopy, **f** Raman spectroscopy, **g** XPS survey spectrum, **h** high-resolution C 1 s spectrum, **i**, **j** TEM images and HRTEM image (inset of Fig. 2j) of CQDs

Figure 2a shows the digital photograph of the industrial Super P powders, and their FESEM images (Fig. S1) reveal that the Super P powders display nanoparticles-like morphology with a size of about 50 nm. As observed from the digital photograph (Fig. S2a), the Super P powders are insoluble in H_2_SO_4_/HNO_3_ mixed solution after the treatment of sonication. However, the digital photograph (Fig. S2b) reveals that the obtained CQDs are highly soluble in H_2_O solution, and also the resulted CQDs aqueous solution does not show any change after 6 months at ambient temperature as manifested in Fig. 2c, suggesting their high stability. Figure 2b is the digital photograph of the final obtained CQDs powders, and the yield is up to 51%. Thus, the scalable synthesis of CQDs with a high yield, together with the advantages of employing a cheap and industrial carbon source, renders them being potential candidate electrode materials for large-scale storage application.

Figure 2d illustrates the XRD pattern of CQDs, and the broad peak of CQDs at around 25° corresponds to (002) plane of the disordered graphite carbon resulting from the strong acid etching [38]. Also, no other peak such as inorganic salts is observed, indicating the high purity of CQDs. FTIR spectrum (Fig. 2e) reveals that the main peaks at 3445, 1720, 1592, 1370 and 1252 cm^−1^ are well fitted to the C–OH, C = O, C = C, C–H and C–O–C bonds [8, 39], respectively, and the existence of these bonds shows that the CQDs are functionalized with the abundant oxygen-containing surface groups, which is particular in favor of the construction of composite materials by the utilization of the functionalized CQDs. Raman spectroscopy (Fig. 2f) was used to further characterize the structure of CQDs. The two obvious peaks at 1346 and 1581 cm^−1^ belong to the D-band and G-band of CQDs, which are attributed to the disordered carbon and ordered graphitic structure, respectively [40]. XPS was undertaken to characterize the elemental composition and carbon bonds of CQDs, as presented in Fig. 2g, h. The four peaks at 284.8, 286.5, 288.4 and 289.2 eV in the spectrum of C1s (Fig. 2h) are, respectively, ascribed to the C–C, C–O, C = O and O–C = O bonds, which accord well with FTIR analysis results [12]. Figure 2i, j exhibits the TEM images of the as-obtained CQDs, and it is clear that the as-prepared CQDs are monodisperse with the uniform distribution in the range of 5–8 nm. As exhibited in the inset of Fig. 2j, the HRTEM image manifests the lattice fringe of 0.215 nm, which accords well with the (100) plane of graphitic carbon, proving the formation of graphitic CQDs.

### Physicochemical and Electrochemical Characterizations of the Cathode Materials

**Fig. 3:**
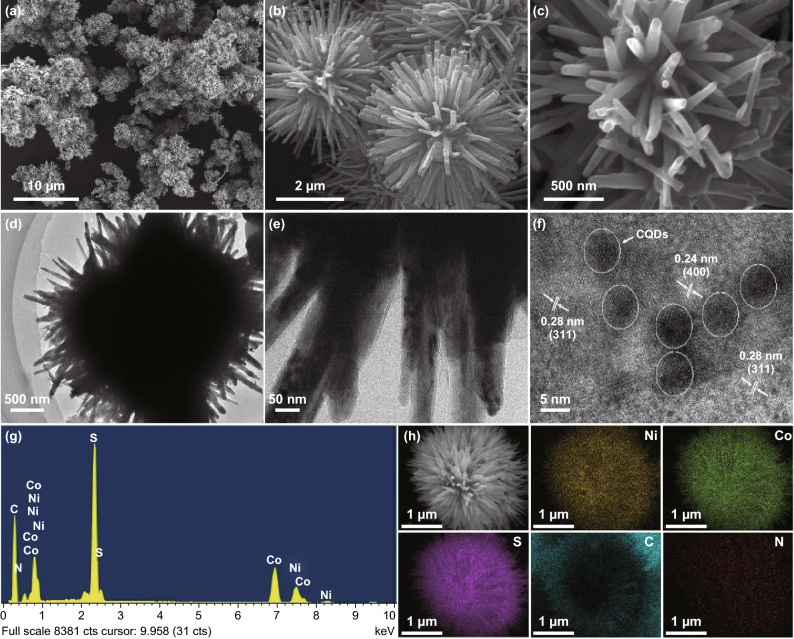
**a**–**c** FESEM images, **d, e** TEM images, **f** HRTEM image, **g** EDS spectrum and **h** corresponding elemental mappings of Ni, Co, S, C and N elements of the N,S-CQDs/NiCo_2_S_4_ composite

The morphology and microstructure of the as-resulted N,S-CQDs/NiCo_2_S_4_ composite were examined by FESEM, TEM, HRTEM, EDS and elemental mappings. As demonstrated in Fig. 3a–c, the FESEM images show that the composite presents the urchin-like microsphere structure with a size of about 3 µm, and the microspheres are composed of numerous nanorods. The superior micro-/nanostructured morphology of the composite is achieved when compared to that of the pristine NiCo_2_S_4_ where only the microspheres with irregular and larger sizes are obtained as exhibited in Fig. S3, which is ascribed to the induced role of CQDs during the formation process of the composite. The micro-/nanostructured morphology of the composite was further surveyed by TEM and HRTEM. The TEM images (Fig. 3d, e) reveal the nanorods-assembled urchin-like microsphere structure with a size of approximately 3 µm, which is well matched with the FESEM images. As displayed in Fig. 3f, the HRTEM image of the composite shows that the CQDs are well combined with NiCo_2_S_4_ to obtain the uniform composite structure. Moreover, the HRTEM image exhibits the lattice fringes of 0.28 and 0.24 nm, which, respectively, accord well with the (311) and (400) planes of NiCo_2_S_4_. In addition, the EDS analysis (Fig. 3g) and corresponding elemental mappings (Fig. 3h) show the existence of Ni, Co, S, C and N, suggesting the formation of N,S-CQDs/NiCo_2_S_4_ composite.

**Fig. 4:**
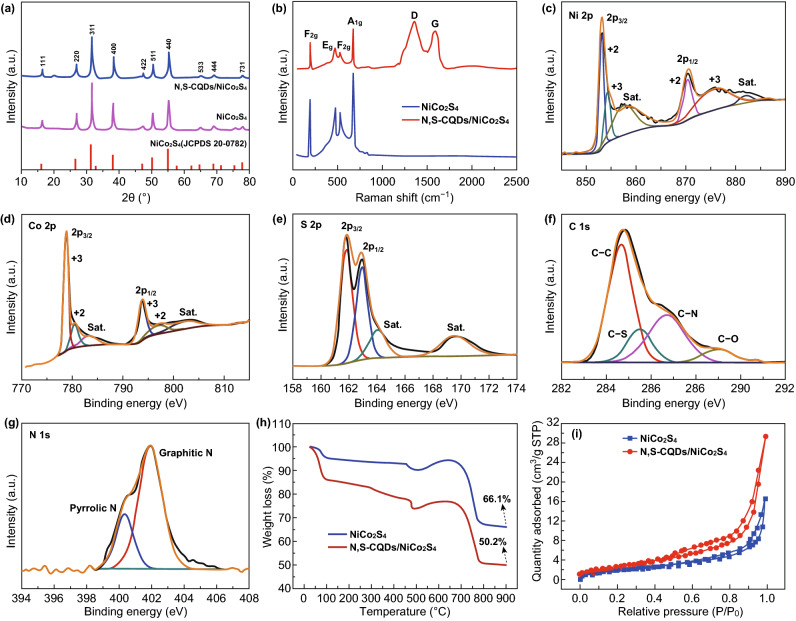
a XRD patterns, **b** Raman spectra of the pristine NiCo_2_S_4_ and N,S-CQDs/NiCo_2_S_4_ composite. XPS high-resolution spectra of **c** Ni 2p, **d** Co 2p, **e** S 2p, **f** C 1s and **g** N 1s of the N,S-CQDs/NiCo_2_S_4_ composite. **h** TG curves and **i** Nitrogen adsorption–desorption isotherms of the N,S-CQDs/NiCo_2_S_4_ composite

XRD was employed to examine the phase and structure of the pristine NiCo_2_S_4_ and N,S-CQDs/NiCo_2_S_4_ composite, and the results are exhibited in Fig. 4a. The main diffraction peaks of the pristine NiCo_2_S_4_ and N,S-CQDs/NiCo_2_S_4_ composite can be clearly observed from the XRD patterns, which all well correspond to the NiCo_2_S_4_ (JCPDS Card No. 20-0782), suggesting that the structure of the composite is not changed in the combination of N,S-CQDs and NiCo_2_S_4_. However, it is hard to notice the peak of N,S-CQDs due to the relatively low content and weak diffraction peak intensity. Raman spectra were used to further characterize the structure of the pristine NiCo_2_S_4_ and N,S-CQDs/NiCo_2_S_4_ composite (Fig. 4b). Four peaks all can be seen for the pristine NiCo_2_S_4_ and N,S-CQDs/NiCo_2_S_4_ composite, which correspond to two *F*_2*g*_ (191 and 524 cm^−1^), one *E*_*g*_ (472 cm^−1^) and one *A*_1*g*_ (675 cm^−1^) Raman modes of NiCo_2_S_4_ [29]. Moreover, the D-band (disordered carbon) and G-band (ordered graphitic carbon) situated at 1356 and 1590 cm^−1^ are characteristic Raman modes of carbon, which are seen from the Raman spectroscopy of the N,S-CQDs/NiCo_2_S_4_ composite.

XPS was further undertaken to study the elemental composition and valence state of the as-synthesized N,S-CQDs/NiCo_2_S_4_ composite, as presented in Fig. 4c–g. The survey spectrum (Fig. S4) demonstrates the coexistence of Ni, Co, S, C, O and N. Figure 4c, d, respectively, exhibits the spectra of Ni 2p and Co 2p. The two peaks situated at 853.04 and 870.44 eV are indexed to Ni^2+^, and another two peaks situated at 854.24 and 876.04 eV are indexed to Ni^3+^, respectively [41]. Similarly, the two peaks situated at 780.54 and 797.24 eV can be ascribed to Co^2+^, and another two peaks situated at 778.74 and 793.79 eV can be attributed to Co^3+^, respectively [41]. The S 2p spectrum presented in Fig. 4e represents two main peaks situated at 161.9 and 163.0 eV, which are, respectively, ascribed to the S^2−^ in low coordination at the surface and the metal–sulfur bonds [34]. For the C 1 s spectrum (Fig. 4f), four main peaks situated at 284.64, 285.49, 286.70 and 289.04 eV are, respectively, assigned to the C–C, C–S, C–N and C–O bonds [24], and the as-formed C–S and C–N bonds indicate that the nitrogen and sulfur elements are successfully codoped into CQDs. Furthermore, Fig. 4g displays the N 1 s spectrum with two main peaks situated at 400.3 and 401.9 eV, which correspond to the pyrrolic N and graphitic N [42]. Based on these results above, the XPS characterization exhibits the coexistence of Ni, Co, S, C and N elements in the N,S-CQDs/NiCo_2_S_4_ composite.

To further determine the composition of the prepared N,S-CQDs/NiCo_2_S_4_ composite, the thermogravimetric analysis (TGA) was executed in air atmosphere from ambient temperature to 900 °C as demonstrated in Fig. 4h. The TG curves of the pristine NiCo_2_S_4_ and N,S-CQDs/NiCo_2_S_4_ composite both show two apparent weight losses and one obvious weight gain. For the N,S-CQDs/NiCo_2_S_4_ composite, the first weight loss before 500 °C is more obvious than that of the pristine NiCo_2_S_4_, which results from the burning of CQDs and the loss of physically adsorbed and crystalline water. The second apparent weight gain between 500 and 650 °C is attributed to the oxidation of NiCo_2_S_4_ to NiSO_4_ and CoSO_4_, and the third evident weight loss in the temperature range of 650 and 800 °C is ascribed to the further burning of CQDs as well as the decomposition of NiSO_4_ and CoSO_4_ to stable NiO and CoO as reported in the literature [43–45]. From the different weight losses of the pristine NiCo_2_S_4_ and N,S-CQDs/NiCo_2_S_4_ composite, it is deduced that the content of CQDs in composite is about 15.9 wt%. N_2_ adsorption/desorption test was employed to research the specific surface area of the pristine NiCo_2_S_4_ and N,S-CQDs/NiCo_2_S_4_ composite, as presented in Fig. 4i. The N,S-CQDs/NiCo_2_S_4_ composite manifests a greater specific surface area (27.2 m^2^ g^−1^) than that of the pristine NiCo_2_S_4_ (14.9 m^2^ g^−1^), which contributes to the enhancement of electrochemical properties.

**Fig. 5:**
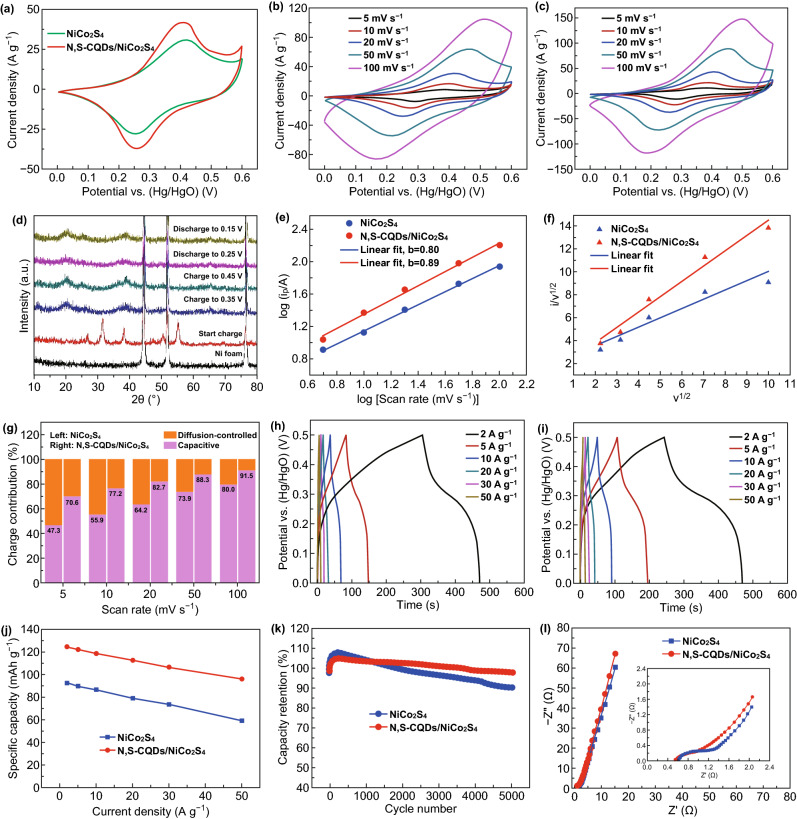
**a** Comparison of CV curves of the pristine NiCo_2_S_4_ and N,S-CQDs/NiCo_2_S_4_ electrodes at 20 mV s^−1^. CV curves at various scan rates of the **b** pristine NiCo_2_S_4_ and **c** N,S-CQDs/NiCo_2_S_4_ electrodes. **d** Ex-situ XRD measurement of the N,S-CQDs/NiCo_2_S_4_ electrode during different electrochemical steps. **e** The fitting lines of log (scan rate, mV s^−1^)-log (i_p_, A), **f** i/ν^1/2^–ν^1/2^, and **g** the charge contribution of the pristine NiCo_2_S_4_ and N,S-CQDs/NiCo_2_S_4_ electrodes. GCD curves at various current densities of the **h** pristine NiCo_2_S_4_ and **i** N,S-CQDs/NiCo_2_S_4_ electrodes. **j** The rate performances, **k** cycling performances, and **l** EIS plots of the pristine NiCo_2_S_4_ and N,S-CQDs/NiCo_2_S_4_ electrodes

The electrochemical performances of the NiCo_2_S_4_ and N,S-CQDs/NiCo_2_S_4_ electrodes were investigated in a three-electrode configuration with 2 M KOH solution as electrolyte. Figure 5a shows the comparison of the CV curves of NiCo_2_S_4_ and N,S-CQDs/NiCo_2_S_4_ electrodes at 20 mV s^−1^ in the potential range of 0 to 0.6 V. The two electrodes both manifest strong and broad redox peaks in the CV curves, implying the combination of Faradaic battery and double-layer capacitive behavior. The Faradaic battery behavior is derived from reversible redox reactions of Ni^2+^/Ni^3+^ and Co^2+^/Co^3+^/Co^4+^ according to Eqs. 1–3 [34]:

1 $${\text{NiS}} + {\text{OH}}^{ - } \leftrightarrow {\text{NiSOH}} + {\text{e}}^{ - }$$

2 $${\text{CoS}} + {\text{OH}}^{ - } \leftrightarrow {\text{CoSOH}} + {\text{e}}^{ - }$$

3 $${\text{CoSOH}} + {\text{OH}}^{ - } \leftrightarrow {\text{CoSO}} + {\text{H}}_{ 2} {\text{O}} + {\text{e}}^{ - }$$

Ex situ XRD measurement was utilized to characterize the phase change of the N,S-CQDs/NiCo_2_S_4_ composite during different electrochemical steps, as exhibited in Fig. 5d. The alteration in reflections indicates the phase change in the charge–discharge process and verifies that Faradaic redox reactions occur in bulk, demonstrating the feature of Faradaic battery. Furthermore, it is clear that the area of CV curve of the N,S-CQDs/NiCo_2_S_4_ electrode is greater than that of the NiCo_2_S_4_ electrode, revealing that the N,S-CQDs/NiCo_2_S_4_ electrode shows higher specific capacity. Figure 5b, c displays the CV curves of the NiCo_2_S_4_ and N,S-CQDs/NiCo_2_S_4_ electrodes at different scan rates, respectively. The N,S-CQDs/NiCo_2_S_4_ electrode manifests a smaller peak position shift than that of the NiCo_2_S_4_ electrode as the scanning speed increases from 5 to 100 mV s^−1^, suggesting that the composite electrode has lower polarization, better conductivity and faster electrochemical kinetic. To further explore the difference of the electrochemical behaviors of the NiCo_2_S_4_ and N,S-CQDs/NiCo_2_S_4_ electrodes, the quantitative analysis was conducted based on the mechanism of charge storage. The relationship of the peak currents (*i*_p_) and scan rates is expressed as *I *= *av*^*b*^ shown in Fig. 5e [26], where the *b*-value of 0.5 and 1 suggests the diffusion-controlled and surface capacitive processes of the electrochemical reaction, respectively. The calculated *b*-value of the NiCo_2_S_4_ and N,S-CQDs/NiCo_2_S_4_ electrodes is, respectively, 0.80 and 0.89, indicating a mixed behavior of diffusion-controlled and surface capacitive processes. The higher *b*-value of the N,S-CQDs/NiCo_2_S_4_ electrode signifies more surface capacitive behaviors. Furthermore, the result is further verified by the quantitative calculation of the contribution of the diffusion-controlled and surface capacitive charge, which is obtained from the following equation [26]: *i*(*V*) = *k*_*1*_*v* + *k*_*2*_*v*^1/2^, in which *k*_*1*_*v* represents the surface capacitive current and *k*_*2*_*v*^1/2^ denotes the diffusion-controlled current. The fitted lines of the *i*/*v*^1/2^ to *v*^1/2^ of the two electrodes are presented in Fig. 5f, and the values of the slope and intercepts, respectively, represent the constants of *k*_*1*_ and *k*_*2*_. The charge contributions of the NiCo_2_S_4_ and N,S-CQDs/NiCo_2_S_4_ electrodes are manifested in Fig. 5g, and it is visible that the N,S-CQDs/NiCo_2_S_4_ electrode displays larger capacitive contribution ratio than that of the NiCo_2_S_4_ electrode at all scan rates, which reasonably elucidates why the N,S-CQDs/NiCo_2_S_4_ electrode possesses faster electrochemical kinetic than that of the NiCo_2_S_4_ electrode.

Figure 5h, i exhibits the GCD curves of the NiCo_2_S_4_ and N,S-CQDs/NiCo_2_S_4_ electrodes at different current densities in the potential window of 0–0.5 V, respectively. The GCD curves of the N,S-CQDs/NiCo_2_S_4_ electrode present a better symmetry than that of the pristine NiCo_2_S_4_ electrode, signifying that the composite electrode has lower resistance and higher rate performance. The specific capacities of the two electrodes are calculated according to the GCD curves, as exhibited in Fig. 5j. The specific capacity of the N,S-CQDs/NiCo_2_S_4_ electrode is, respectively, 124.4, 122.4, 118.6, 112.8, 106.4 and 96.1 mAh g^−1^ at 2, 5, 10, 20, 30 and 50 A g^−1^, which is better than that of the NiCo_2_S_4_ electrode (from 92.8 mAh g^−1^ at 2 A g^−1^ to 59.1 mAh g^−1^ at 50 A g^−1^). Correspondingly, the N,S-CQDs/NiCo_2_S_4_ electrode manifests a greater capacity retention of 77.3% at 50 A g^−1^ than that of the NiCo_2_S_4_ electrode (63.7% of capacity retention at 50 A g^−1^). The improved specific capacity and rate performance of the N,S-CQDs/NiCo_2_S_4_ electrode originate from the positive synergistic effect between highly conductive N,S-CQDs with the capacitive feature and high-capacity NiCo_2_S_4_ with Faradaic characteristic. Note that the specific capacity and rate performance of the as-obtained N,S-CQDs/NiCo_2_S_4_ composite are comparable or even higher than those of nickel cobalt sulfide-based composite as reported in the literature [46–53]. The cycling property of the NiCo_2_S_4_ and N,S-CQDs/NiCo_2_S_4_ electrodes was surveyed by the repeated GCD tests at 5 A g^−1^ in the potential range of 0–0.5 V over 5000 cycles, as exhibited in Fig. 5k. The specific capacity of the two electrodes increases in the first hundreds of cycles due to the gradually sufficient activation process and then reduces with a gradual decline as the increasing cycles [23, 26]. After 5000 cycles, the N,S-CQDs/NiCo_2_S_4_ electrode manifests a better cycle property with 97.9% of the initial capacity retention than that of the NiCo_2_S_4_ electrode (90.3%), revealing the excellent cycling stability of the as-resulted composite electrode. Additionally, the EIS test was utilized to illustrate the superior electrochemical behaviors, which was implemented in the frequency range of 0.01 Hz to 100 kHz at the open-circuit potential (Fig. 5l). The Nyquist plot consists of the intercept and depressed semicircle in the high-frequency region as well as the slope line in the low-frequency region [54, 55]. The N,S-CQDs/NiCo_2_S_4_ electrode displays smaller intercept, lower semicircle diameter and higher slope value than those of the NiCo_2_S_4_ electrode, indicating the lower resistance and better electrical conductivity of the composite electrode, leading to the improved electrochemical properties.

### Physicochemical and Electrochemical Characterizations of the Anode Materials

**Fig. 6:**
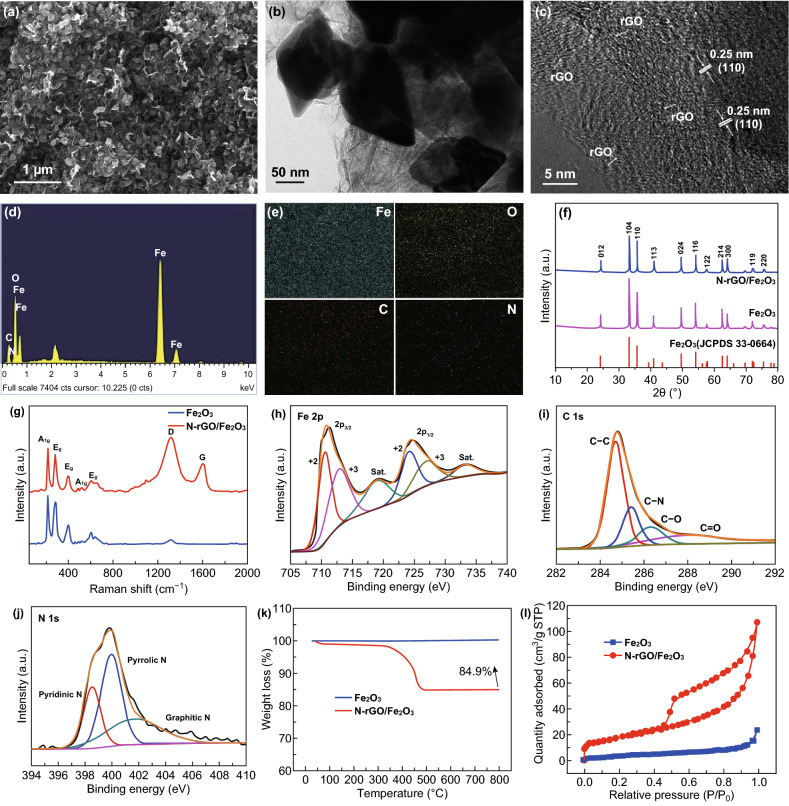
**a** FESEM image, **b** TEM image, **c** HRTEM image, **d** EDS spectrum and **e** corresponding elemental mappings of Fe, O, C and N elements of the N-rGO/Fe_2_O_3_ composite. **f** XRD patterns and **g** Raman spectra of the pristine Fe_2_O_3_ and N-rGO/Fe_2_O_3_ composite. XPS high-resolution spectra of **h** Fe 2p, **i** C 1 s and **j** N 1s of the N-rGO/Fe_2_O_3_ composite. **k** TG curves and **l** nitrogen adsorption–desorption isotherms of the N-rGO/Fe_2_O_3_ composite

Figures 6a and S5 display the FESEM images of the N-rGO/Fe_2_O_3_ composite, and it is clearly observed that the prism-like Fe_2_O_3_ hexahedrons are tightly wrapped by the N-rGO layers, suggesting the close contact between the Fe_2_O_3_ hexahedrons and the N-rGO sheets. Such a composite structure can increase their contact and hinder the agglomeration of Fe_2_O_3_ and N-rGO, thereby promoting the improvement of electrochemical activity and stability of the composite. TEM study demonstrated in Figs. 6b and S6 was employed to verify the formation of the N-rGO/Fe_2_O_3_ composite, and the prism-like Fe_2_O_3_ hexahedrons with a size of about 100 nm were well combined with N-doped rGO sheets to form the composite. It is noted that the pristine Fe_2_O_3_ displays the morphology of prism-like hexahedrons from the FESEM and TEM images demonstrated in Figs. S7 and S8, indicating that the morphology of the composite is not altered in the combination of Fe_2_O_3_ and N-rGO. As exhibited in Fig. 6c, the HRTEM image indicates the presence of crumpled N-rGO, and the lattice fringe of 0.25 nm matches well with the (110) crystal plane of Fe_2_O_3_. EDS and the corresponding elemental mappings were utilized to examine the elemental composition and distribution of the N-rGO/Fe_2_O_3_ composite. The EDS spectrum (Fig. 6d) verifies the existence of Fe, O, C, and N in the composite, and elemental mappings (Fig. 6e) reveal that these elements are uniformly distributed throughout the composite.

XRD was utilized to further characterize the structure and phase of the pristine Fe_2_O_3_ and N-rGO/Fe_2_O_3_ composite (Fig. 6f). The XRD patterns show the strong diffraction peaks of the pristine Fe_2_O_3_ and N-rGO/Fe_2_O_3_ composite, which are both well fitted to the Fe_2_O_3_ (JCPDS Card No. 33-0664). Note that the characteristic peaks of rGO cannot be observed in the XRD pattern of the composite, which results from the weak and broad peak and low rGO content in composite. Raman spectra were used to further examine the structure of the pristine Fe_2_O_3_ and N-rGO/Fe_2_O_3_ composite (Fig. 6g). Five peaks all can be seen from the Raman spectra of the pristine Fe_2_O_3_ and N-rGO/Fe_2_O_3_ composite, which are attributed to two *A*_1*g*_ (218 and 483 cm^−1^) and three *E*_*g*_ (283, 399, and 601 cm^−1^) Raman modes of Fe_2_O_3_ [56]. Besides, two typical peaks situated at 1320 and 1596 cm^−1^ are assigned to the D-band and G-band, respectively, further suggesting that the Fe_2_O_3_ is successfully combined with the N-rGO to form the composite.

XPS was further applied to explore the elemental composition and valence state of the obtained N-rGO/Fe_2_O_3_ composite, as presented in Fig. 6h–j. The survey spectrum (Fig. S9) demonstrates the existence of Fe, O, C and N in the N-rGO/Fe_2_O_3_ composite. For the Fe 2p spectrum (Fig. 6h), the peaks situated at 710.5 and 724.1 eV are assigned to Fe^2+^, and the peaks situated at 712.8 and 726.8 eV are well fitted to Fe^3+^ [57]. As for the C 1 s spectrum shown in Fig. 6i, the peaks located at 284.7, 285.4, 286.3 and 288.1 eV correspond to the C–C, C–N, C–O and C = O bonds [58], and the as-formed C–N bond implies that the nitrogen element is well doped into rGO. Furthermore, the N 1 s spectrum manifested in Fig. 6j reveals that the peaks located at 398.5, 399.9 and 401.6 eV correspond well with the pyridinic N, pyrrolic N and graphitic N [59]. Thus, the XPS analysis further proves the formation of the N-rGO/Fe_2_O_3_ composite.

The content of GO in N-rGO/Fe_2_O_3_ composite was determined by TGA test, which is executed in air atmosphere from room temperature to 800 °C as demonstrated in Fig. 6k. From the TGA curves, almost no weight loss of the pristine Fe_2_O_3_ can be seen in the whole temperature range, suggesting the high stability of the Fe_2_O_3_ in air, while an obvious weight loss of the N-rGO/Fe_2_O_3_ composite can be observed between 350 and 470 °C due to the burning of rGO. Apparently, the rGO content in the N-rGO/Fe_2_O_3_ composite is deduced to be about 15.1 wt%. As exhibited in Fig. 6l, the BET specific surface areas of the pristine Fe_2_O_3_ and N-rGO/Fe_2_O_3_ composite are obtained by the nitrogen adsorption/desorption test. Benefiting from the introduction of N-rGO in composite, the N-rGO/Fe_2_O_3_ composite displays a far greater BET specific surface area (66.8 m^2^ g^−1^) than that of the pristine Fe_2_O_3_ (13.8 m^2^ g^−1^), and thus, it is expected that the N-rGO/Fe_2_O_3_ composite can present excellent electrochemical performances.

**Fig. 7:**
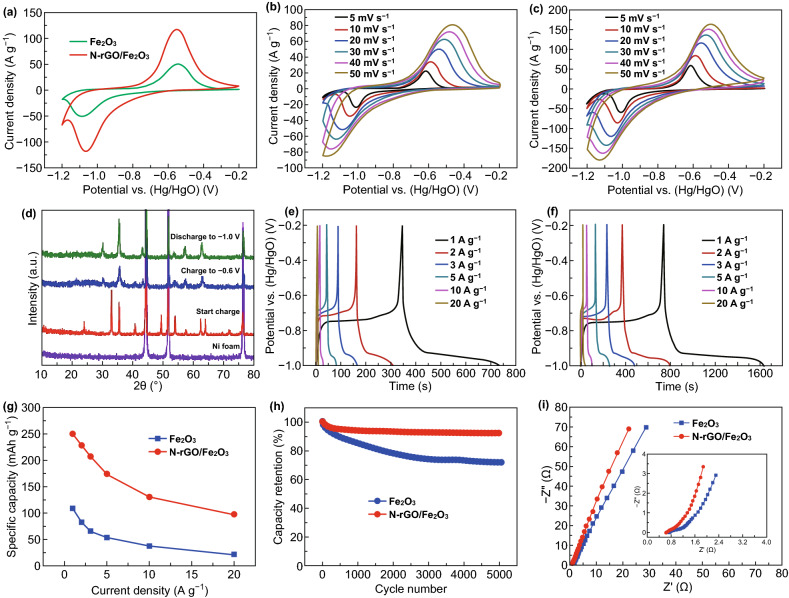
**a** Comparison of CV curves of the pristine Fe_2_O_3_ and N-rGO/Fe_2_O_3_ electrodes at 20 mV s^−1^. CV curves at various scan rates of the **b** pristine Fe_2_O_3_ and **c** N-rGO/Fe_2_O_3_ electrodes. **d** Ex-situ XRD measurement of the N-rGO/Fe_2_O_3_ electrode during different electrochemical steps. GCD curves at various current densities of the **e** pristine Fe_2_O_3_ and **f** N-rGO/Fe_2_O_3_ electrodes. **g** The rate performances, **h** cycling performances, and **i** EIS plots of the pristine Fe_2_O_3_ and N-rGO/Fe_2_O_3_ electrodes

The electrochemical properties of the pristine Fe_2_O_3_ and N-rGO/Fe_2_O_3_ composite were also studied in a three-electrode cell with 2 M KOH solution as electrolyte. Figure 7a presents the comparison of CV curves of the Fe_2_O_3_ and N-rGO/Fe_2_O_3_ electrodes at 20 mV s^−1^ within the potential range of − 1.2 to− 0.2 V. Apparently, the composite electrode manifests a distinctly greater area of CV curve than that of the Fe_2_O_3_ electrode, implying that the composite electrode shows much larger specific capacity than that of the pristine Fe_2_O_3_ electrode. The substantially improved specific capacity of the composite electrode is ascribed to the positive synergistic effect of the double-layer capacitive behavior of N-rGO and Faradaic redox reaction of Fe_2_O_3_. Ex situ XRD test (Fig. 7d) was also used to investigate the phase change of the N-rGO/Fe_2_O_3_ composite during different electrochemical steps. The observed change in reflections reveals the phase change in the charge/discharge process and confirms the occurring of Faradaic redox reactions, further demonstrating the typical battery characteristic. In comparison with the Fe_2_O_3_ electrode, the N-rGO/Fe_2_O_3_ electrode shows a smaller potential difference of the anodic and cathodic peak (∆E_P_), implying the lower resistance and better kinetic behavior. Figure 7b, c displays the CV curves of the Fe_2_O_3_ and N-rGO/Fe_2_O_3_ electrodes, respectively, and no distinct distortion can be noticed with the increasing scanning speed from 5 to 50 mV s^−1^. Figure 7e, f exhibits the GCD curves of the Fe_2_O_3_ and N-rGO/Fe_2_O_3_ electrodes at various current densities in the potential range between− 1 and − 0.2 V, respectively. The calculated specific capacity of the N-rGO/Fe_2_O_3_ electrode (Fig. 7g) is, respectively, 249.6, 228.4, 206.7, 174.2, 130.4 and 96.2 mAh g^−1^ at 1, 2, 3, 5, 10 and 20 A g^−1^, which is much better than that of the Fe_2_O_3_ electrode (from 109.1 at 1 A g^−1^ to 21.8 mAh g^−1^ at 20 A g^−1^). Moreover, the specific capacity of the N-rGO/Fe_2_O_3_ electrode is not only far higher than that of carbon-based anode materials, but also comparable to or even larger than that of most Fe_2_O_3_-based anode materials reported in the literature [56–58, 60–64]. The cycle property of the Fe_2_O_3_ and N-rGO/Fe_2_O_3_ electrodes was further compared by the repeated GCD tests at 3 A g^−1^ over 5000 cycles, as manifested in Fig. 7h. The N-rGO/Fe_2_O_3_ electrode manifests a better cycle stability with 92.1% capacity retention than that of the Fe_2_O_3_ electrode (71.8%) after 5000 cycles, suggesting that the unique N-rGO-tightly-wrapped Fe_2_O_3_ composite structure can prevent the agglomeration of Fe_2_O_3_ and N-rGO and alleviate the volume change in the repeated charging-discharging processes, leading to the improvement of cycle performance. Moreover, the EIS measurement was taken, and the Nyquist plots are demonstrated in Fig. 7i. The N-rGO/Fe_2_O_3_ electrode shows smaller intercept and semicircle diameter in the high-frequency region as well as higher slope value in the low-frequency region than those of the Fe_2_O_3_ electrode. Therefore, the results well demonstrate why the N-rGO/Fe_2_O_3_ electrode presents improved electrochemical performances in comparison with the Fe_2_O_3_ electrode.

### Electrochemical Characterizations of the N,S-CQDs/NiCo_2_S_4_//N-rGO/Fe_2_O_3_ Alkaline Aqueous Battery

**Fig. 8:**
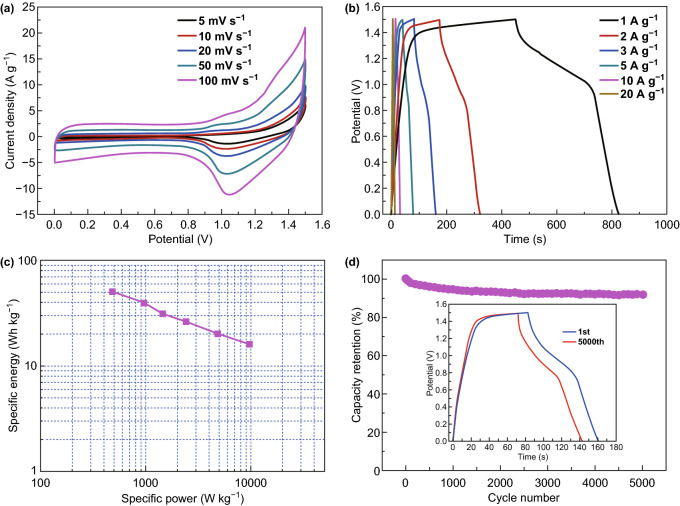
**a** CV curves, **b** GCD curves, **c** Ragone plots and **d** cycling performance of the N,S-CQDs/NiCo_2_S_4_//N-rGO/Fe_2_O_3_ device

To further clarify the superior electrochemical properties of the N,S-CQDs/NiCo_2_S_4_ and N-rGO/Fe_2_O_3_ composites, a novel alkaline aqueous battery was assembled by, respectively, employing the two composites as the cathode and anode, and the electrochemical performances, including CV, GCD and EIS, were tested in a voltage window of 0–1.5 V with 2 M KOH solution as electrolyte. Figure 8a displays the CV curves of the as-obtained alkaline aqueous battery at scan rates ranging from 5 to 100 mV s^−1^, and the shape of CV curves indicates that the capacity originates from the combined contribution of both Faradaic redox reaction and capacitive EDLC at all scan rates. The GCD curves of the alkaline aqueous battery at current densities of 1-20 A g^−1^ are presented in Fig. 8b, and the as-obtained result is further employed to investigate the specific energy (*E*) and specific power (*P*) of the alkaline aqueous battery according to the following two formulas [10]: *E* = 0.5*C*(∆*V*)^2^/3.6 and *P* = *E*/*t*, where the *C*, ∆*V* and *t* are, respectively, the specific capacity, voltage difference and discharge time of the alkaline aqueous battery. As demonstrated in Ragone plot of Fig. 8c, it is found that the alkaline aqueous battery delivers a superior specific energy of 50.2 Wh kg^−1^ at a specific power of 485 W kg^−1^ and still maintains a decent specific energy of 16.1 Wh kg^−1^ even at an ultrahigh specific power of 9.7 kW kg^−1^, implying the excellent power capability. Moreover, the highest specific energy of the assembled alkaline aqueous battery in our work is comparable to or even higher than that of alkaline aqueous devices reported in the literature, including NiCo_2_O_4_/CQDs//AC [10], NiCo_2_S_4_/NCF//OMC/NCF [32], Ni–Co–S/G//PCN [34], CuCo_2_O_4_/CuO//Fe_2_O_3_/rGO [56], MnO_2_/FGS//Fe_2_O_3_/FGS [60], Ni(OH)_2_//AC [65], CF/NiCo_2_S_4_@PPy//CF/MoS_2_ [66], NiCo_2_S_4_@G//PC [67], Ni-Co-S@G//RGH [68] and MnO/NCA//Fe_2_O_3_/NCA [69]. Additionally, the cycling property of the as-obtained device demonstrated in Fig. 8d was estimated by the GCD measurements at 3 A g^−1^ over 5000 cycles, and 91.5% of capacity retention is maintained, suggesting the superior cycle stability.

The superior electrochemical properties of the N,S-CQDs/NiCo_2_S_4_//N-rGO/Fe_2_O_3_ device are attributed to the following factors: (1) The utilization of CQDs as structure-directing agents induces the formation of unique N,S-codoped CQDs-decorated urchin-like nanorods-assembled NiCo_2_S_4_ microspheres composite. The nanosized building blocks of the composite with a large surface area afford more efficient electroactive sites for charge storage, contributing to the enhanced specific capacity, and the micrometer-sized assemblies of the composite possess desirable and mechanical properties and superior structure stability, leading to the improved cycling life. Meanwhile, the utilization of CQDs as conductive agents, together with the N,S-codoped modification, greatly enhances the electronic conductivity of the composite, leading to the improvement of rate property; (2) the unique prism-like nanostructured Fe_2_O_3_ hexahedrons can provide sufficient surface active sites for redox reaction, giving rise to the enhanced specific capacity, and the conductive N-rGO-decorated Fe_2_O_3_ can significantly boost the electrical conductivity of the composite anode materials, facilitating the fast electron transport kinetic and resulting in the enhanced rate property. Moreover, the Fe_2_O_3_ hexahedrons are tightly wrapped by the N-rGO layers, which effectively prevents the agglomeration and buffer against the volume change during the charging/discharging cycle process, substantially improving the cycling performance; (3) the ultrahigh-capacity N-rGO/Fe_2_O_3_ composite anode materials are utilized to couple with the high-performance N,S-CQDs/NiCo_2_S_4_ composite cathode materials to fabricate the alkaline aqueous battery, and the rational design and selection of cathode and anode materials render the resulting device achieve the enhanced specific energy, ultrahigh specific power and superior cycling life.

## Conclusions

In a word, the graphitic CQDs are prepared via a scalable mixed-acid chemical oxidation method employing highly conductive and low-cost Super P powders as precursors, and the high yield of more than 50% is successfully acquired. Then, the resulting CQDs are further used as both structure-directing and conductive agents to controllably synthesize the N,S-codoped CQDs-decorated urchin-like NiCo_2_S_4_ composite microsphere cathode materials for the first time, which display evidently improved electrochemical properties including specific capacity, rate performance and cycle life benefited from the synergistic effect of highly conductive N, S-codoped CQDs and unique micro-/nanostructured NiCo_2_S_4_ with Faradaic redox feature. Meanwhile, the N-rGO-wrapped prism-like Fe_2_O_3_ hexahedrons composite anode materials are prepared, which exhibit an ultrahigh specific capacity, substantially enhanced rate performance and cycle property due to the synergistic effect between highly conductive N-rGO and high-capacity nanostructured Fe_2_O_3_ hexahedrons. In view of their superior electrochemical properties and rational coupling of cathode and anode materials, a novel alkaline aqueous battery is assembled by employing N,S-CQDs/NiCo_2_S_4_ as cathode and N-rGO/Fe_2_O_3_ as anode in 2 M KOH aqueous electrolyte. The fabricated N,S-CQDs/NiCo_2_S_4_//N-rGO/Fe_2_O_3_ device delivers a high specific energy of 50.2 Wh kg^−1^ at a specific power of 485 W kg^−1^ or an ultrahigh specific power of 9.7 kW kg^−1^ at a decent specific energy of 16.1 Wh kg^−1^ and good cycling life with 91.5% of capacity retention at 3 A g^−1^ over 5,000 cycles. The present research gives a valuable reference for the design and exploitation of superior energy storage devices using battery/capacitive composite materials simultaneously as both cathode and anode in aqueous electrolyte.

## Electronic Supplementary Material

Below is the link to the electronic supplementary material.

Supplementary material 1 (PDF 644 kb)
