# Supplementary material for: Graphitic Carbon Quantum Dots Modified Nickel Cobalt Sulfide as Cathode Materials for Alkaline Aqueous Batteries
Source: Nanomicro Lett. 2020 Jan 4;12:16. doi: 10.1007/s40820-019-0355-0 (PMC7770733; doi:10.1007/s40820-019-0355-0)
Supplement: Supplementary file 1 — Supplementary material 1 (PDF 644 kb) [file 40820_2019_355_MOESM1_ESM.pdf]

Supporting Information for

Graphitic Carbon Quantum Dots Modified Nickel Cobalt Sulfide as Cathode Materials for Alkaline Aqueous Batteries

Yirong Zhu[1], Jingying Li[1], Xiaoru Yun[1], Ganggang Zhao[2], Peng Ge[2], Guoqiang Zou[2], Yong Liu[3], Hongshuai Hou[2,]*, Xiaobo Ji[2]

[1]College of Metallurgy and Material Engineering, Hunan University of Technology, Zhuzhou 412007, People's Republic of China

[2]College of Chemistry and Chemical Engineering, Central South University, Changsha 410083, People's Republic of China

[3]State Key Lab of Powder Metallurgy, Central South University, Changsha 410083, People's Republic of China

*Corresponding author. E-mail: hs-hou@csu.edu.cn (Hongshuai Hou)

Supplementary Figures

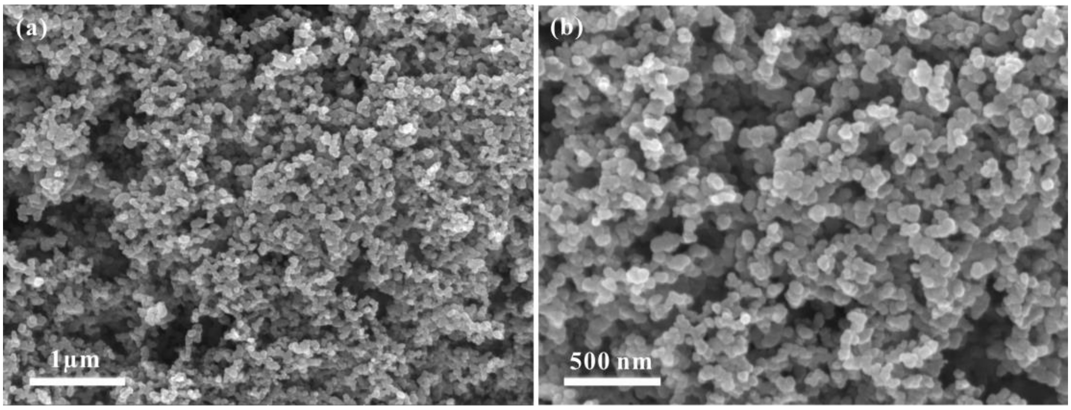

Fig. S1 FESEM images of Super P powders with different magnification

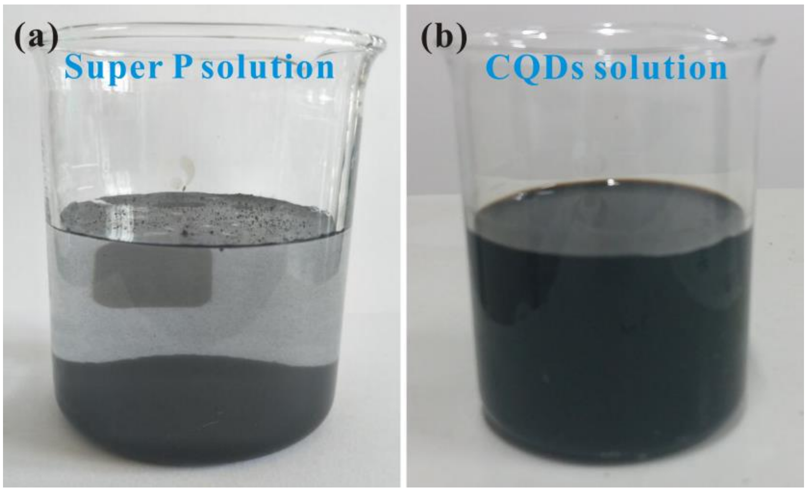

Fig. S2 Digital photographes of **a** Super P powders in H_2SO_4/HNO_3 aqueous solution, and **b** CQDs aqueous solution

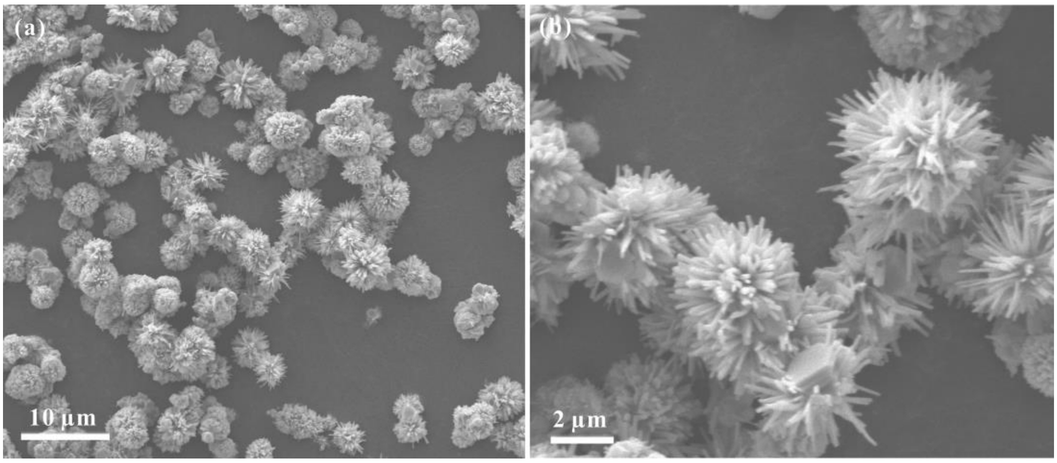

Fig. S3 FESEM images of the pristine $NiCo_2S_4$ with different magnification

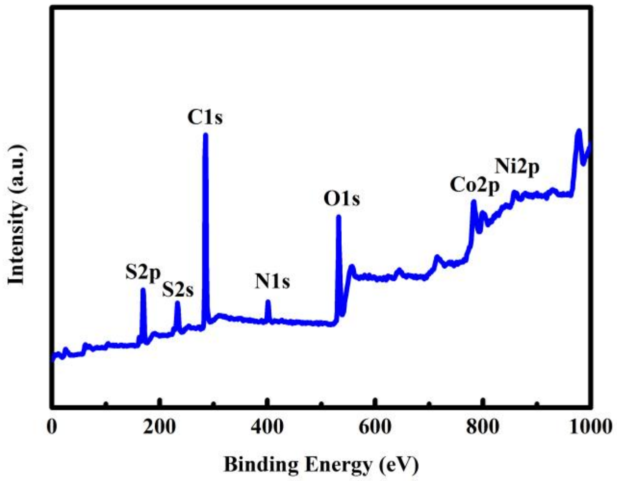

Fig. S4 XPS survey spectrum of the N,S-CQDs/$NiCo_2S_4$ composite

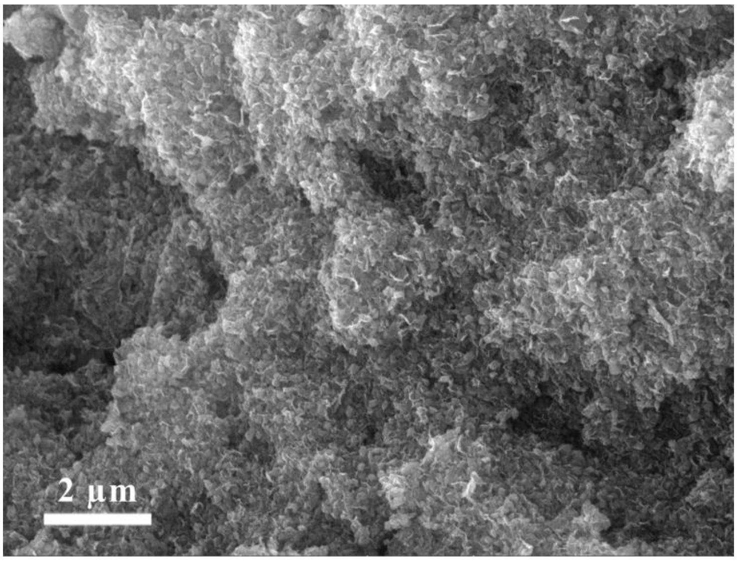

Fig. S5 FESEM image of the N-rGO/Fe_2O_3 composite

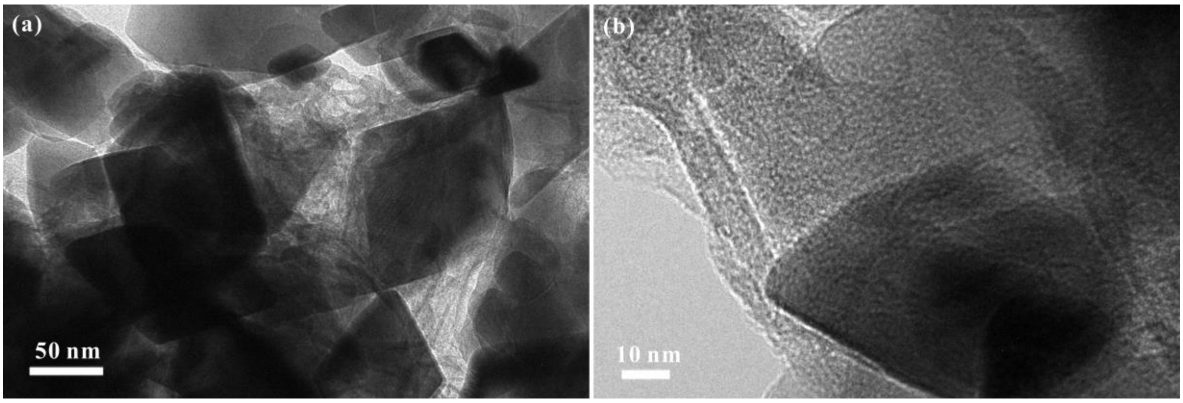

Fig. S6 TEM images of the N-rGO/Fe_2O_3 composite with different magnification

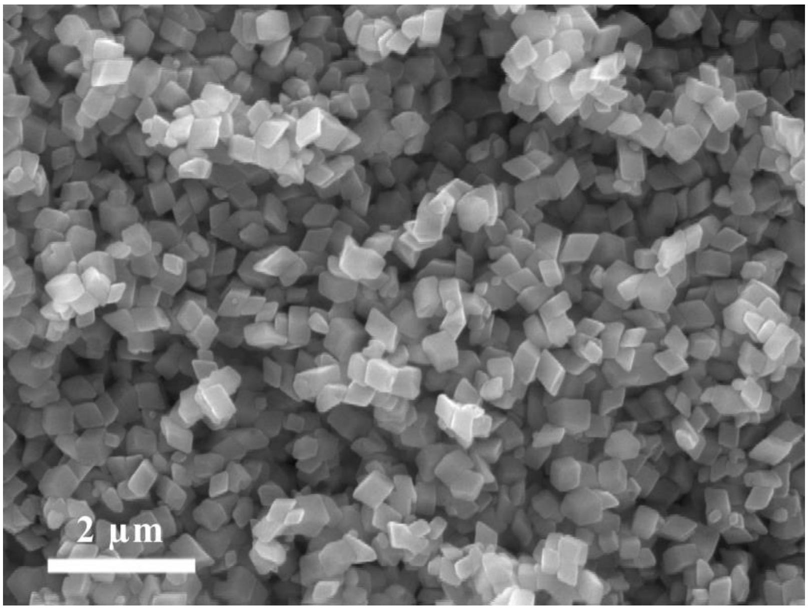

Fig. S7 FESEM image of the pristine Fe_2O_3

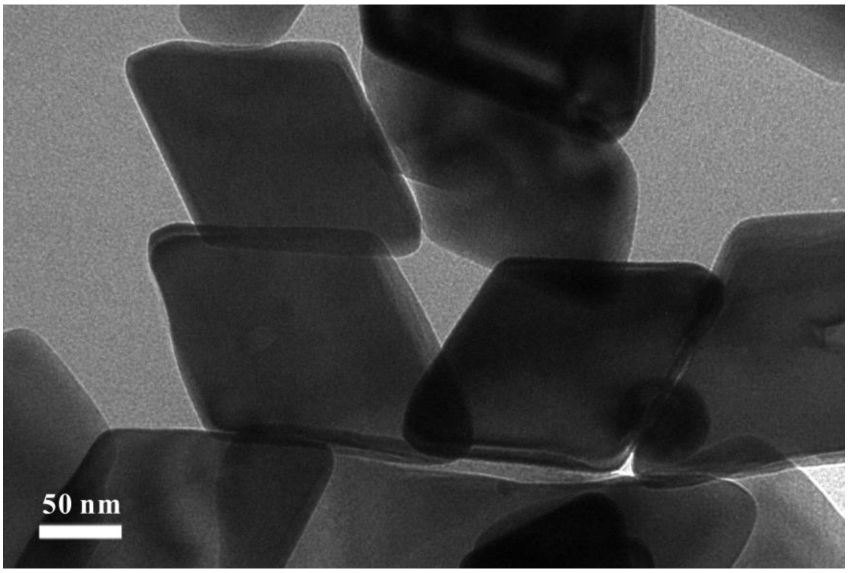

Fig. S8 TEM image of the pristine Fe_2O_3

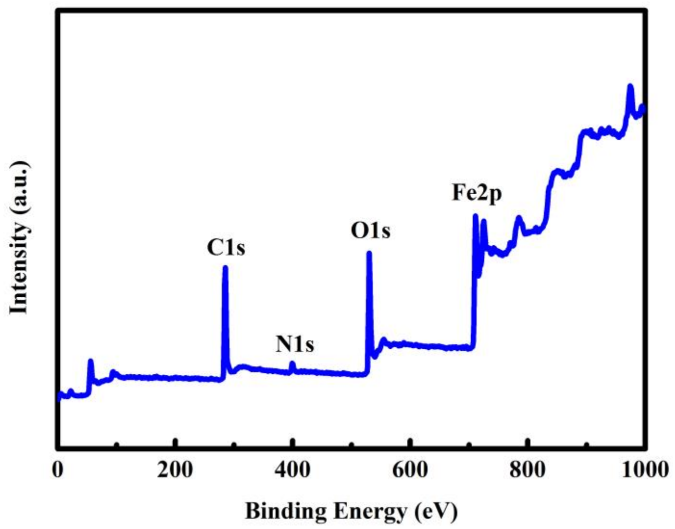

Fig. S9 XPS survey spectrum of the N-rGO/Fe_2O_3 composite
